# Two QTLs controlling Clubroot resistance identified from Bulked Segregant Sequencing in Pakchoi (*Brassica campestris* ssp. *chinensis* Makino)

**DOI:** 10.1038/s41598-019-44724-z

**Published:** 2019-06-25

**Authors:** Hongfang Zhu, Wen Zhai, Xiaofeng Li, Yuying Zhu

**Affiliations:** Shanghai Key Lab of Protected Horticultural Technology, Horticultural Research Institute, Shanghai Academy of Agricultural Sciences, Shanghai, 201106 China

**Keywords:** Genetic markers, DNA sequencing, Genome duplication

## Abstract

Clubroot, caused by *Plasmodiophora Brassicae*, is a serious soil-borne disease in worldwide. In recent years, progression of clubroot is rapid and serious in Shanghai, China. In this study, The inheritance of clubroot resistance (CR) were determined in pakchoi using F_2_ segregation population that were developed by crossing highly resistant line ‘CR38’ and susceptible line ‘CS22’. Two novel QTLs, *qBrCR38-1* and *qBrCR38-2*, was identified by BSA-seq (Bulked Segregant Sequencing) resistant to *P. brassicae* physiological race 7. Two significant peak *qBrCR38-1* and *qBrCR38-2* were observed by three statistical methods between interval of 19.7–20.6 Mb in chromosome A07 and 20.0–20.6 Mb in chromosome A08, respectively. In addition, Polymorphic SNPs identified within target regions were converted to kompetitive allele-specific PCR (KASP) assays. In target regions of *qBrCR38-1* and *qBrCR38-2*, there were twenty SNP sites identified, eleven KASP markers of which are significantly associated to CR (P < 0.05). Seven candidate genes were identified and found to be involved in disease resistance (TIR-NBS-LRR proteins), defense responses of bacterium and fungi and biotic/abiotic stress response in the target regions harboring the two QTLs. Two novel QTLs and candidate genes identified from the present study provide insights into the genetic mechanism of CR in *B.rapa*, and the associated SNPs can be effectively used for marker-assisted breeding.

## Introduction

Clubroot, caused by the obligate endoparasite *Plasmodiophora Brassicae*, is recognized as a serious soil-borne disease. It infects cruciferous Brassica oil crops and vegetables and is specially associated with considerable yield losses^1–3^. The pathogen causes abnormal growth of the plant and eventually leads to a massive gall formation on the root^4^. Clubroot prevents the transportation of water and nutrients, causing the plant to wilt and finally dies off. In recent years, the incidence of clubroot in the suburbs of Shanghai, China, has gradually become increasingly more serious. Until 2017, 39 counties and 9 towns of Shanghai had a breakout of clubroot disease and the affected area had reached 2500 hm2. Pathotypes of *P. brassicae* was physiological race 7 in Qingpu distract of Shanghai. Clubroot disease is difficult to completely prevent and control by cultural practices or chemical treatments such as anti-microbial compounds or crop rotations^5,6^. Therefore, development of resistant cultivars is the most effective approach to control clubroot disease.

In *Brassica Rapa*, genetic analysis and quantitative trait locus (QTL) mapping studies have identified at least eight race-specific clubroot resistance(CR) loci. *Crr1, Crr2, and Crr4* CR genes from European turnip cv ‘Siloga’, which were mapped onto chromosomes A08, A01, and A06, respectively^7,8^. The resistance genes *CRa* from turnip line ‘ECD02’^9^, *CRb* from turnip line ‘Gelria R’^10,11^ and *Crr3* from turnip cv. “Milan White”^12,13^ were both located on the chromosome A03. *CRk* and *CRc* were identified from turnip cv. “Debra” line ECD01 and ECD02, mapped onto A03 and A02, respectively^14,15^. In *B. rapa* canola, *Rcr4*, *Rcr8*, and *Rcr9* breeding line T19 were mapped to chromosomes A03, A02, and A08, respectively through genotyping by sequencing^16^. The resistance gene *Rcr1* from pak choy cv. “Flower Nabana” was also mapped to A03 by transcriptome analysis, and two candidate genes *Bra019409* and *Bra019410* were screened for correlation with clubroot-resistance^17^. Meanwhile, clubroot-resistant gene *Rcr2* was also mapped on chromosome 3 in chinese cabbage ‘Jazz’. Five SNP markers co-segregated with *Rcr2* were developed between 22 to 26 Mb, and *Bra019410* and *Bra019413* are most likely candidates of *Rcr2*, with conserved domain of TIR-NBS-LRR resistance protein^18^.

Bulked segregant analysis by sequencing (BSA-seq), is an effective technique used to identify quantitative trait loci (QTLs)^19,20^. It is a genomics tool used for genetic mapping which takes advantage of bulked-segregant analysis and high-throughput genotyping using next-generation sequencing (NGS)^21,22^. BSA-Seq has been applied to mapping agronomically important loci in Arabidopsis, rice and wheat^23–25^. Candidate genes for disease resistance have been identified successfully by this approach, such as a broad-spectrum resistance gene Pi65 (t) in rice^26^, downy mildew and powdery mildew resistance QTLs in cucumber^27,28^. BSA-seq technologies, have proven successful for rapidly establishing the association of agronomic traits with molecular markers and had a major impact on crop breeding^29^. Meanwile, different statistical methods for poolsd QTL mapping have been proposed, including MutMap^23^, G’ value^30^ and ED^31^, that would have noise reduction and highlighting effect of QTLs. Moreover, it requires an efficient platform for applying molecular markers to marker-assisted selection (MAS) in breeding. Kompetitive Allele Specific PCR (KASP) is one of the high-throughput SNP genotyping technologies is a cost-effective, low genotyping error rates and flexible system which is widely used for genetic mapping, trait-specific markers development, germplasm characterization (genetic diversity, genetic relationship, and population structure), and quality control (QC) analysis (genetic identity, genetic purity, and parentage verification)^32–34^.

The Pakchoi (*Brassica campestris* ssp. *chinensis* Makino), also called non-heading Chinese cabbage, is one of the most important Brassica vegetable crop in China and East Asia^35^. Most Pakchoi cultivars are highly susceptible to the *P*. *brassicae*. In this study, BSA-seq was applied to determine the inheritance of clubroot resistance (CR) in Pakchoi inbred line ‘CR38’ and F_2_ population. Then, the underlying QTLs were mapped by three statistical methods and examined sequence variation in the target region to identify the most probable candidate gene associated with CR.

## Results

### Clubroot Resistance evaluations in CR38 × CS22

**Figure 1:**
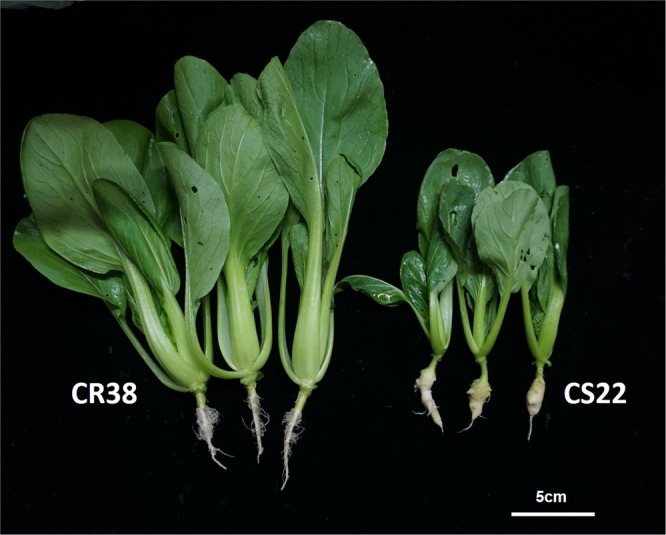
phenotype of the parents: CR38 (Clubroot resistance) and CS22 (Clubroot susceptible). Plants were inoculated with 7th physiology race of *P. brassicae*.

‘CR38’ were highly resistant (DSI = 3.33 in 2016, DSI = 4.76 in 2017) to the 7 physiology race of *P. brassicae* in contrast to the ‘CS22’ which was more susceptible (DSI = 100 in 2016, DSI = 94.44 in 2017). ‘CR38’ and ‘CS22’ (Fig. 1a), were crossed to develop segregating populations for QTL analysis of CR. Clubroot symptoms of 294 F_2_ individuals were identified in autumn 2017. F_2_ population exhibited a continuous frequency distribution with a range of 0–3 and DSI of 34.24, suggesting polygenic control of CR in this population (Table 1).

**Table 1:** Disease evaluations of CR in CR38 × CS22.

Parents	Type	Year	Disease rating					DSI
			0	1	2	3	Total	
CR38	R	2016	27	3	0	0	30	3.33
		2017	18	2	0	0	20	4.76
CS22	S	2016	0	0	0	15	15	100.00
		2017	0	0	4	18	22	94.44
CR38 × CS22	F2	2017	81	145	43	23	292	34.24

$${\rm{DSI}}( \% )=\frac{\sum ({\rm{rating}}\,{\rm{class}})\times (\#{\rm{plant}}\,{\rm{in}}\,{\rm{rating}}\,{\rm{class}})\,}{({\rm{total}}\,\#\,{\rm{plants}}\,{\rm{in}}\,{\rm{treatment}})\times 3}\times 100$$.

### Whole genome sequencing analysis

BSA-seq were used to identify the loci controlling CR in our F_2_ population, from which 22 highly resistant and 22 highly susceptible individuals were selected and two DNA pool were created. Illumina high-throughput sequencing generated 51,292,360 and 59,493,747 short reads (150 bp in length) from the CR-pool and the CS-pool, with a coverage of 98.11% (39-fold genome coverage) and 98.46% (45-fold genome coverage), respectively. Over 97% of the reads in both pools were mapped to the Chiifu-401 reference genome. The Q20% was 96.12% and 95.91% of the CR -pool and CS-pool. The effective sequencing depths for CR38 and CS22 were 20-fold and 25-fold genome coverage, respectively, which guaranteed accuracy of the subsequent analysis. The results from sequencing are presented in Table 2.

**Table 2:** Summary of BSA sequencing data for each sample.

Sample Name	Clean Reads	Clean Base(G)	Clean Q20 (%)	Clean GC Content (%)	coverage rate (%)	Map reads rate (%)	Effective Rate (%)	Sequencing depth
CR-pool	51,292,360	15.39	96.12	39.62	98.11%	97.03%	99.35	39×
CS-pool	59,493,747	17.85	95.91	39.16	98.46%	97.07%	99.56	45×
CR38	27,262,388	8.18	97.75	39.91	91.89%	97.11%	98.99	20×
CS22	32,499,790	9.75	96.08	39.8	94.09%	97.08%	99.26	25×

### Mapping of Clubroot resistance

**Figure 2:**
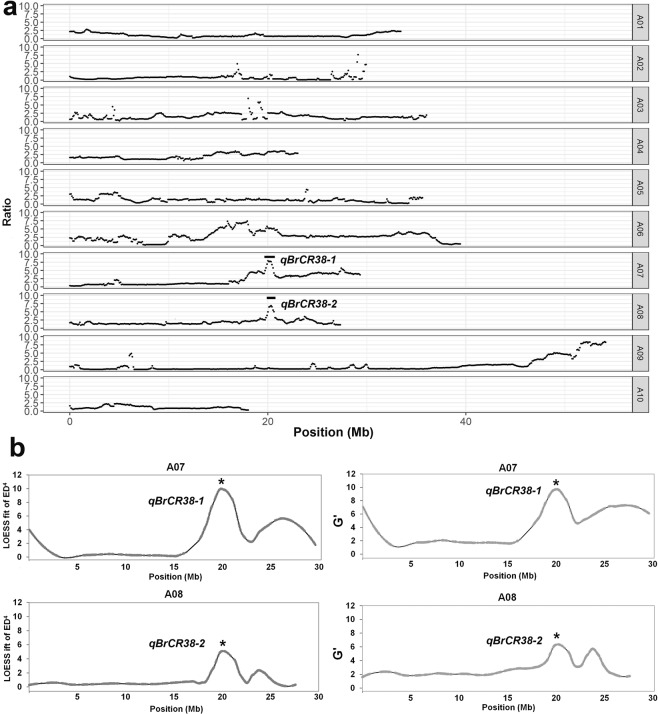
Genome-wide scan for clubroot resistance QTLs using BSA-seq. Two significant QTL of *qBrCR38-1* and *qBrCR38-2* detected in chromosomes A07 and A08 using three statistical approach. (**a**) Ratio (CR-pool/CS-pool) of the SNP-ratios (Resistant alleles/sensitive alleles) is presented. The significance peak is indicated by the horizontal dotted line. (**b**) Distribution of G’value and Loess fit curve calculated using ED^4^ data on chromosome A07 and A08

Sequence data were trimmed and filtered prior to analysis. Compared to the reference genome, 1,489,940 SNPs were identified between the CR38 and CS22 parents. Based on the uniquely mapped reads, association analysis between the two bulks was performed on 1,079,828 SNPs. To identify genomic regions associated with CR, three methods were used to mapping the QTLs. Firstly, Scanning each SNP in the whole genome, the Ratio of SNP-ratioin CR-pool and CS-pool was calculated^36^ (Fig. 2A). Two significant peak *qBrCR38-1* and *qBrCR38-2* was observed between 19.7–20.6 Mb of chromosome A07 and 20.0–20.6 Mb of chromosome A08 (Fig. 2a). Next, Allelic segregation between CR and CS pools by Euclidean Distance (ED)^31^ were measured. ED^4^ was raising ED to the fourth power to decrease noise. Local polynomial regression methods (LOESS fit) of ED^4^ calculated shown in Fig. 2b. The identified peaks that have an ED above a threshold and a high allele frequency in the CR-pool. G value at each SNP were calculated, and G is a smooth version of G^30^. The statistics result demonstrated that there were two QTLs at the same peaks position about 20.1 Mb in chromosome A07 and 20.2 Mb in chromosome A08 (Fig. 2b). We found 5441 and 1887 SNPs between the two parents in the target regions of chromosome A07 and chromosome A08.

**Figure 3:**
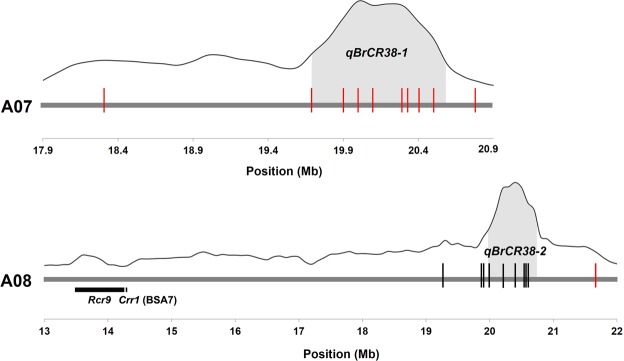
Close-up view of *qBrCR38-1* and *qBrCR38-2* and KASP markers link to the target regions of CR. Vertical lines represent the position of KASP markers, where red lines were significant (P < 0.05) associated with CR. Gray areas is the target regions.

Corresponding sequences in flanking 50 bp region were used to develop allele-specific KASP primers which were genotype of the parental lines and F_2_ population. There were twenty SNP sites identified at physical positon 18.4 Mb – 20.8 Mb and 19.4 Mb – 21.8 Mb around regions of *qBrCR38-1* and *qBrCR38-2* (Table 3). *qBrCR38-1* was mapped in the interval of 0.35 cM flanked by Br_K_07106 and Br_K_07107. Br_K_080103 and Br_K_080107 are linkage to *qBrCR38-2* within the genetic distance of 0.33 cM. To evaluate the effect of *qBrCR38-1* and *qBrCR38-2*, F_2_ population were classified into two groups (AA and aa) based on the genotype of the most tightly linked markers. Lines carrying the CR38 alleles (AA) are significantly (P < 0.05) clubroot resistant than those carrying the CS22 alleles (aa) in eleven SNP markers (Table 3). Close-up view of QTLs and KASP markers link to the target regions was shown in Fig. 3.

**Table 3:** KASP marker in target region.

Marker Name	QTL	Chr	Target region (Mb)	Position (bp)	P value
Br_K_070101	*qBrCR38-1*	A07	19.7–20.6	18391107	0.027*
Br_K_070113	*qBrCR38-1*	A07	19.7–20.6	19759488	0.026*
Br_K_070105	*qBrCR38-1*	A07	19.7–20.6	19995501	0.008**
Br_K_070106	*qBrCR38-1*	A07	19.7–20.6	20107291	0.003**
Br_K_070107	*qBrCR38-1*	A07	19.7–20.6	20209491	0.002**
Br_K_070115	*qBrCR38-1*	A07	19.7–20.6	20333647	0.002**
Br_K_070109	*qBrCR38-1*	A07	19.7–20.6	20389706	0.002**
Br_K_070110	*qBrCR38-1*	A07	19.7–20.6	20447844	0.003**
Br_K_070116	*qBrCR38-1*	A07	19.7–20.6	20501616	0.009**
Br_K_070103	*qBrCR38-1*	A07	19.7–20.6	20813113	0.008**
Br_K_080101	*qBrCR38-2*	A08	20.0–20.6	19389881	0.345
Br_K_080112	*qBrCR38-2*	A08	20.0–20.6	19918702	0.240
Br_K_080115	*qBrCR38-2*	A08	20.0–20.6	19936667	0.328
Br_K_080118	*qBrCR38-2*	A08	20.0–20.6	20067617	0.356
Br_K_080107	*qBrCR38-2*	A08	20.0–20.6	20254038	0.484
Br_K_080109	*qBrCR38-2*	A08	20.0–20.6	20444233	0.531
Br_K_080111	*qBrCR38-2*	A08	20.0–20.6	20608800	0.652
Br_K_080120	*qBrCR38-2*	A08	20.0–20.6	20628063	0.536
Br_K_080121	*qBrCR38-2*	A08	20.0–20.6	20638194	0.652
Br_K_080103	*qBrCR38-2*	A08	20.0–20.6	21748451	0.048*

*P < 0.05, **P < 0.01.

### Candidate gene identification

A total of 188 and 116 annotated genes encoded sequences encompassed the target regions of *qBrCR38-1* and *qBrCR38-2*, respectively. The functional annotation of genes in the target region indicates their potential involvement in fungal disease resistance assessed for further analysis. In chromosome A07, candidate gene *BraA07002412*, homologous to *AT1G80460*, encodes protein *NHO1* (*nonhost resistant gene 1*) similar to glycerol kinase and performs a rate-limiting step in glycerol metabolism. In addition, candidate gene *BraA08002455* in chromosome A08, homologous to *AT1G65850*, encodes Toll-Interleukin-1 receptor/nucleotide binding site/leucine-rich repeat (TIR-NBS-LRR class) disease resistance protein. Moreover, six genes with F-box domain (*BraA0700224*9, and *BraA07002494* in A07, *BraA08002451* and *BraA08002452*in A08) and a WRKY transcription factor (*BraA08002471*) were considered as the functional genes associated with CR (Table 4).

**Table 4:** Candidate gene annotation.

Gene ID	Chr	Position	*A. thaliana* homologs	Annotations
BraA07002249	A07	19177733–19181091	AT4G02310	F-box family protein
BraA07002412	A07	19975263–19977367	AT1G80460	NHO1 (nonhost resistance gene.)
BraA07002494	A07	20387426–20389115	AT1G78750	F-box family protein
BraA08002451	A08	19715762–19716810	AT4G39756	F-box family protein
BraA08002452	A08	19719445–19720378	AT4G39753	F-box family protein
BraA08002455	A08	19759513–19760286	AT1G65850	disease resistance protein (TIR-NBS-LRR class)
BraA08002471	A08	19872938–19875271	AT4G39410	WRKY transcription factor

## Discussion

**Figure 4:**
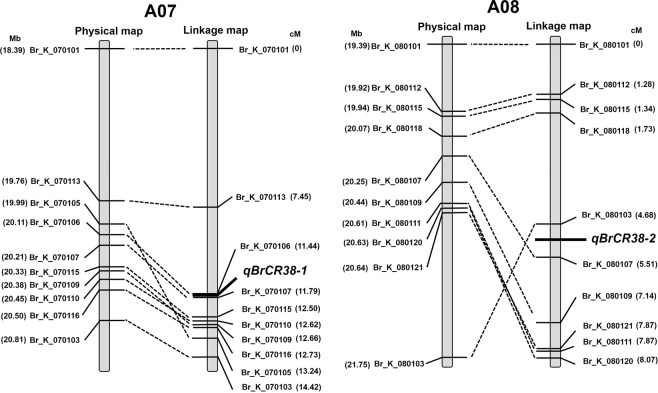
The physical map corresponds to the genetic map around region of *qBrCR38-1* and *qBrCR38-2*. Broken lines drawn regions defined by KASP markers on chromosome A07 and A08. Physical locations are on the left, and the genetic distance is shown on the right.

The traditional fine mapping method is time-consuming and is rather laborious to require the identification of genome-wide polymorphic DNA markers for linkage analysis. Furthermore, it needs to use the genetic mapping and physical mapping to locate the target gene in the specific location of chromosomes. With the rapid development of sequencing technology and genomics, using BSA-seq of extreme mixed pools, a large number of traits can be located quickly and effectively. Compared to traditional QTL mapping, BAS-seq has the advantages of simple operation and short test period, and does not require specific near-isogenic lines to be constructed. Through high-throughput sequencing, thousands of SNPs can be captured at one time, and this variation information can be used to identify trait-related genes or QTLs as well as to develop molecular markers. The accuracy of BSA-seq results is very important to identify the resistance loci of CR, and there are many influencing factors. With improvement of reference genome in Chinese cabbage using NGS and Pacbio SMRT sequencing, it is possible to map genes for different traits in Chinese cabbage crops effectively and get more complete gene sequences. In order to highlight the significance of the positioning interval for better noise reduction, Three appropriate statistical approach were used to observed same two significant peak of *qBrCR38-*1 and *qBrCR38-2*. SNP-ratio and ED4 increasing CR sensiticity near the high allele frequency locus in the CR-pool. G’ is expected to decrease much more rapidly around the causal site, implying narrower intervals of support around QTLs^30^. BSA-Seq combination with KASP analysis is a powerful approach for fine mapping of causal genes. KASP is a cost-effective single-step genotyping technology, cheaper than SSRs and more flexible than genotyping by sequencing (GBS) or array-based genotyping. In our research, KASP markers linkaged CR were obtained in the target region of QTLs. Alignments of the physical map corresponds to the genetic map around target regions were showed good collinearity (Fig. 4). The inconsistencies such as Br_K_080103 might be caused by chromosomal rearrangement in Pakchoi. Eleven SNP sites were significantly associated to CR, but nine SNP sites have week effective to CR in chromosome A08. Thus it is speculated that *qBrCR38-*1 is a major effective locus. In general, our research provide an important way to molecular marker assisted selection for CR on backcross breeding and gene pyramiding in *B.rapa*.

Several recent studies reported that there were at least eight pathotypes of *P. brassica* pathogen. Pathotypes 2, 3, 4, 5, 6, 7, 10, 11 represent the main races of *P. brassicae* pathogen in China as classified by Williams identification system^37,38^. The clubtoot-resistant genes *Crr1*, *Crr2* and *CRb* have resistance to physiological race 4^7,8^. *Rcr1*-*Rcr9* have high resistance to pathotypes 2, 3, 5, 6 and 8 in Canada^17,18,39^. In the present study, the resistant parent “CR38” has high resistance to *P. brassicae* of physiological race 7 which is widespread in Shanghai, China. There were no clubroot resistant genes, even no resistant cultivars. Therefore, the development of cultivars and the mapping genes resistant to physiological race 7 in Pakchoi will provide a basis for disease resistance breeding of Chinese cabbage.

Previous researches have applied several strategies to identify and map genes and QTL involved in CR in *B.rapa. Crr1* and *Rcr9*^16^ were both located in chromosomes A08, while no genes or QTLs were found in chromosomes A07. In the present study, clubroot resistance in ‘CR38’ was identified as quantitative inheritance and was regulated by multiple genes. In addition, *qBrCR38-1* and *qBrCR38-2* in chromosomes A07 and A08 associated with CR in Pakchoi were detected by BSA-seq. We blasted the sequence from linkage marker (BSA7) of *Crr1* and *Rcr9* target region to *B.rapa* reference genome V2.5. *Crr1* and *Rcr9* were located in 14.27 Mb and 13.5–14.2 Mb respectively, thus showing that these two gene were not in the same chromosomal position as *qBrCR38-2* (Fig. 3).

Annotation information and homologous sequence alignment of candidate genes revealed new disease-resistant genes involved in CR and further studies are required. NOH1 was reported to be related to both general and specific resistance against bacterium and fungi^40^. TIR-NBS-LRR resistant protein plays an important role in regulating the activity of plant disease resistance proteins^41^. Recent studies suggested that F-box proteins may up-regulate defense-related gene expression in rice^42^, and play a role in cell death and defense responses activated during pathogen recognition in tobacco and tomato^43^. WRKY transcription factors family is known to be involved in biotic/abiotic stress response^44^. Overall, Genomic intervals of QTL loci still need to be narrowed down to determine candidate genes. Candidate genes identified from the present study provide insights into the genetic mechanism of clubroot resistant in *B.rapa*.

In conclusion, BSA-seq Combine with KASP analysis was carried out to conduct the mapping of CR in Pakchoi. As a result, two novel QTLs regions on chromosome A07 and A08 respectively were detected, and seven genes in target chromosome region associated with the disease resistance were considered as candidates for CR. These genes need to be further studied, and the associated SNPs should be used for marker-assisted breeding of CR in *B.rapa*

## Materials and Methods

### Plant materials and phenotypic data collection

Two Pakchoi inbred line, CR38 (Clubroot resistance) and CS22 (Clubroot susceptible), were used as parents to generate 292 F_2_ individuals for studies on inheritance and genetic mapping. CR38 is cold tolerant type and highly resistant to the 7th physiology race of *Plasmodiophora Brassicae* by using the inoculation method of Williams (1966)^45^. The pathogen was propagated on Pakchoi, and the clubs in infected roots were stored at −20 °C until required. All CR test plants were sown in a pot containing 5 × 10^6^ spores per gram of dry soil, and cultivation was carried out in a growth chamber at 25/20 °C (day/night) with a photo-period of 14 h. At 6 weeks after sowing, the root symptoms of each plant were evaluated. A disease severity index (DSI) of two parents was calculated as according to Suwabe (2003)^8^. Each test included control-resistant and susceptible cultivars. All plant materials examined in this study were obtained from Shanghai academy of agricultural science.

### Library construction and Whole genome sequencing

Total genomic DNA from the two parental lines, CR38 and CS22, was isolated from young leaves according to the CTAB method^46^. Equal amounts of genomic DNA were sampled from individuals with leaves (22 DNA samples for each extreme trait) and bulked to generate the Clubroot Resistance pool (CR-pool) and Clubroot Sensitive pool (CS-pool). Pair-end sequencing libraries with a read length of 150 bp and insert sizes of approximately 350 bp were subjected to whole-genome re-sequencing with Illumina HiSeq 2500. Short reads obtained from both parents and two DNA-bulks were aligned against the *B. rapa* Chiifu-401 reference genome sequence v2.5 (http://brassicadb.org/brad/) to obtain the consensus sequence using BWA software^47^. Reads of CR-pool and CS-pool were separately aligned to CR38 and CS22 consensus sequence reads to call SNPs with GATK tools software^48^. Heterozygote alleles in two parents were filtered out during the process. Raw read data are archived at the NCBI Sequence Read Archive (SRA) under Accession PRJNA497673.

### QTL mapping and target region annotation

Assuming that “A” is a SNP identified between CR38 and CS22, the CR38 and CS22 genotypes at this site are “AA” and “aa”, respectively. For the CR-pool, nRA and nRa are the numbers of reads containing “A” and “a”, Respectively. Three methods were used to mapping QTLs. Firstly, SNP-ratio (Resistant alleles/sensitive alleles) of CR-pool and CS-pool were calculated. Then, CR-pool SNP-ratio was divided by CS-pool SNP-ratio and plotted across the genomic regions that showed ratio peaks, which indicate the possible existence of a QTL^36^

$${Ratio}=({\rm{nRA}}/{\rm{nRa}})/({\rm{nSA}}/{\rm{nSa}})$$

Read depth for each allele at segregating allelic SNPs in 500 kb sliding windows was summed using a 100 kb step increment.

The ED for each SNP was calculated according to a previous report^31^. ED is the sum of 100 ED-SNP values within a window of 100 consecutive SNPs. ED^4^ was then calculated by raising ED to the fourth power. Loess fit curves for SNP allele frequency ED^4^. G’, the G value averaged across neighboring SNPs, was calculated as described by Magwene *et al*. (2011)^30^. There methods for mapping common target regions were considered as significant loci.

A list of genes within the target regions was generated using the *B.rapa* genome annotation data (http://brassicadb.org/brad/), and these genes were used to query the GO annotation on the website of gene ontology to identify putative resistance related genes for further analysis.

### KASP Marker development and genotyping in target region

Polymorphic SNPs identified within the target regions for clubroot resistance were converted into KASP markers (Supplementary Table S1). Each SNP site were parsed to retrieve the flanking sequences 50 bp for KASP markers in the target region. The criteria for selection were that the flanking sequences (a) did not contain any other SNP and InDels, (b) had no more than four consecutive repeats, (c) was close to the ratio peaks on the physical location and (d) Primer sequences were relatively unique in the genome.

For each SNP, two allele-specific forward primers and one common reverse primer were designed using the Primer3 software. Target region SNPs were converted into KASP primers and were used to test the entire F2 population. Reactions were performed in 384 well plates, with a final reaction volume of 3 μl, which contained 1.48 μl of KASP 2X reaction mix, 50 ng of template DNA, 0.17 μM Hex forward primer, 0.17 μM FAM forward primer and 0.42 μM universal reverse primer. The following cycling conditions were used: 15 min at 94 °C followed by 10 touchdown cycles of 20 s at 95 °C and 60 s at 65 °C (dropping 0.8 °C per cycle); after the final annealing temperature of 56 °C was achieved, there were 26 cycles of 20 s at 94 °C and 60 s at 57 °C. Thermocycling and fluorescence readings were performed on Hydrocycler and PHERAstar of LGC SNPline platform. Genotyping data viewed as a cluster plot by SNPviewer software supported from LGC Genomics (http://www.lgcgenomics.com). The significance of the correlation coefficients between genotype and phenotype was determined with t-tests. Linkage groups were performed with JoinMap 4.1 (Van Ooijenand Voorrips, 2001).

## Supplementary information

Primer sequence of KASP marker
