# Supplementary material for: Two QTLs controlling Clubroot resistance identified from Bulked Segregant Sequencing in Pakchoi (Brassica campestris ssp. chinensis Makino)
Source: Sci Rep. 2019 Jun 25;9:9228. doi: 10.1038/s41598-019-44724-z (PMC6592919; doi:10.1038/s41598-019-44724-z)
Supplement: Supplementary file 1 — Primer sequence of KASP marker [file 41598_2019_44724_MOESM1_ESM.doc]

**Two QTLs controlling Clubroot resistance identified from Bulked Segregant Sequencing in Pakchoi** **(*Brassica campestris* ssp.**

***chinensis* Makino)**

Hongfang Zhu*, Wen Zhai*, Xiaofeng Li, and Yuying Zhu^#^

Shanghai Key Lab of Protected Horticultural Technology, Horticultural Research Institute, Shanghai Academy of Agricultural Sciences, Shanghai 201106, China

^*^Hongfang Zhu and Wen Zhai contributed equally to this work.

^#^Correspondence should be addressed to Y. Zhu (email: yy5@saas.sh.cn)

| **Marker Name** | **Allele X** | **Allele Y** | **Position** | **Primer Seq Allele X** | **Primer Seq Allele Y** | **Primer Seq Common** |
| --- | --- | --- | --- | --- | --- | --- |
| BR_K_070101 | G | T | A07.18391107 | CCAACTGAATCTTCATCGTC | GCCAACTGAATCTTCATCGTA | TCGACAACGACTACCATCAC |
| BR_K_070113 | T | C | A07.19759488 | CAAAATCTGTGTCAGGAGGT | CAAAATCTGTGTCAGGAGGC | CGGATACACCCGTATCTACC |
| BR_K_070105 | G | A | A07.19995501 | GATGGTAAATGAGAAGGGAAG | GATGGTAAATGAGAAGGGAAA | TAGTGGATATCACCCCTATGC |
| BR_K_070106 | G | T | A07.20107291 | GGTTTCAATGAACTGAATCTTTC | GGTTTCAATGAACTGAATCTTTA | CAGCTCATATGTTGCTGACA |
| BR_K_070107 | G | T | A07.20209491 | TATTGCATACTTCTGTCGGG | AGTATTGCATACTTCTGTCGGT | TCGGTATGTGATATTCGTGTC |
| BR_K_070115 | T | C | A07.20333647 | CCAGTGGTCCTGGAGTGA | CCAGTGGTCCTGGAGTGG | AGCAATAGGAAGCAGCTCAT |
| BR_K_070109 | A | C | A07.20389706 | AGTCGTCACGTCTAGGAGA | AGTCGTCACGTCTAGGAGC | AGTGTGATCCGAGGGATG |
| BR_K_070110 | G | T | A07.20447844 | GCTATCAATTTGCTTCGTGG | AGCTATCAATTTGCTTCGTGT | TTCATGAGCATACCTCCAAA |
| BR_K_070116 | T | C | A07.20501616 | CTGTTTCGATTAATCTGTCTCATAA | CTGTTTCGATTAATCTGTCTCATAG | CCCGAAAATTTGTTGATCTT |
| BR_K_070103 | G | T | A07.20813113 | AATATGGTAACATGCGGAAAG | AATATGGTAACATGCGGAAAT | TGCATTTATACGTTTTTGTTG |
| BR_K_080101 | A | C | A08.19389881 | AATTTGACCCCAGAAAAGTT | AATTTGACCCCAGAAAAGTG | CTTTCTTGCTCCCTACTCCA |
| BR_K_080112 | T | C | A08.19918702 | AGGGTCGCAGTGGTCAAT | AGGGTCGCAGTGGTCAAC | GAAGCTTAGCTATGGCCTCA |
| BR_K_080115 | G | T | A08.19936667 | CAGAAGAACCAGACAACCAC | CAGAAGAACCAGACAACCAA | GTGGCTGGTAATCGGAGAT |
| BR_K_080118 | A | T | A08.20067617 | CCTTGAGTTAAGTATGAAAGAAAAA | CCTTGAGTTAAGTATGAAAGAAAAT | TTTTCTCATGAGTATAACCTCTATTG |
| BR_K_080107 | A | T | A08.20254038 | AAAACTATCATTCACCAAAAAACT | AAAACTATCATTCACCAAAAAACA | ATACCAATGGAGCTCGAACA |
| BR_K_080109 | G | A | A08.20444233 | AGCACTTTTGTACTCCCTCC | AGCACTTTTGTACTCCCTCT | CGTGCATATGTGTAAAACGAC |
| BR_K_080111 | G | T | A08.20608800 | CGCACGTGACAGACATTC | ACCGCACGTGACAGACATTA | CTACGTCGTTCCACTGACAA |
| BR_K_080120 | G | A | A08.20628063 | TTCATCTGACTCGTAATCACC | CATTCATCTGACTCGTAATCACT | GTTCTTGAGCCCAATCTGAC |
| BR_K_080121 | G | A | A08.20638194 | GCTCCAGTACTCTTCAACCTC | GCTCCAGTACTCTTCAACCTT | AACTTGAGAGGCTTGTCTTGA |
| BR_K_080103 | G | A | A08.21748451 | GTGTCATGTTTGGTTTGGC | TGTGTCATGTTTGGTTTGGT | AAAGAGAAGGCATAAGAGTTTAAGA |

Supplementary Table S1：Primer sequence of KASP marker
